# Supplementary material for: Integrated Logic Circuits Based on Wafer-Scale 2D-MoS2 FETs Using Buried-Gate Structures
Source: Nanomaterials (Basel). 2023 Oct 30;13(21):2870. doi: 10.3390/nano13212870 (PMC10649149; doi:10.3390/nano13212870)
Supplement: Supplementary file 1 [file nanomaterials-13-02870-s001.zip › nanomaterials-2637622-supplementary.pdf]

nanomaterials

Article

Integrated Logic Circuits Based on Wafer-scale 2D-MoS_2 FETs using Buried-Gated Structures

Ju-Ah Lee [1,2,†], Jongwon Yoon [1,†], Seungkwon Hwang [1], Hyunsang Hwang [3], Jung-Dae Kwon [1], Seung-Ki Lee [2,*] and Yonghun Kim [1,*]

[1] Department of Energy and Electronic Materials, Surface Materials Division, Korea Institute of Materials Science (KIMS), Changwon 51508, Republic of Korea; jualee0208@kims.re.kr (J.A.L.); jwyoon@kims.re.kr (J.Y.); hhs0505@kims.re.kr (S.H.); jdkwon@kims.re.kr (J.-D.K.)
[2] School of Materials Science and Engineering, Pusan National University, Busan 46241, Republic of Korea
[3] Center for Single Atom-Based Semiconductor Device, Department of Materials Science and Engineering, Pohang University of Science and Technology (POSTECH), Pohang 37673, Republic of Korea; hwanghs@postech.ac.kr
* Correspondence: ifriend@pusan.ac.kr (S.-K.L.); kyhun09@kims.re.kr (Y.K.); Tel.: +82-(0)55-280-3281 (Y.K.)
† These authors contributed equally to this work.

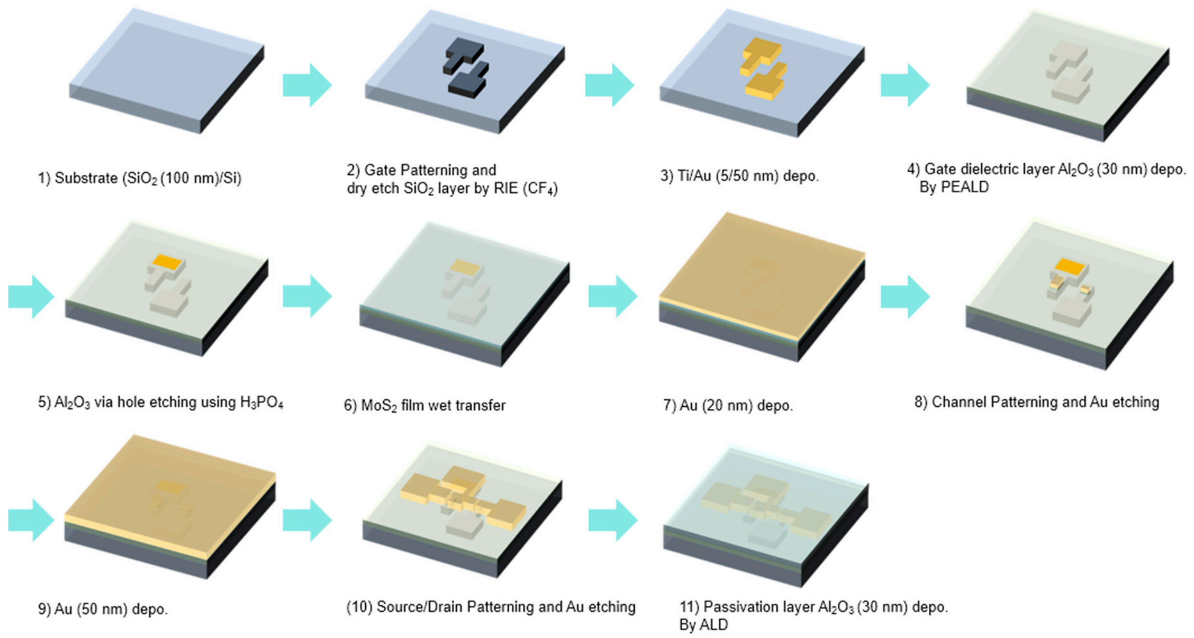

Figure S1. The schematic of fabrication flow for the 2D-MoS_2 inverter based on buried-gate structure.

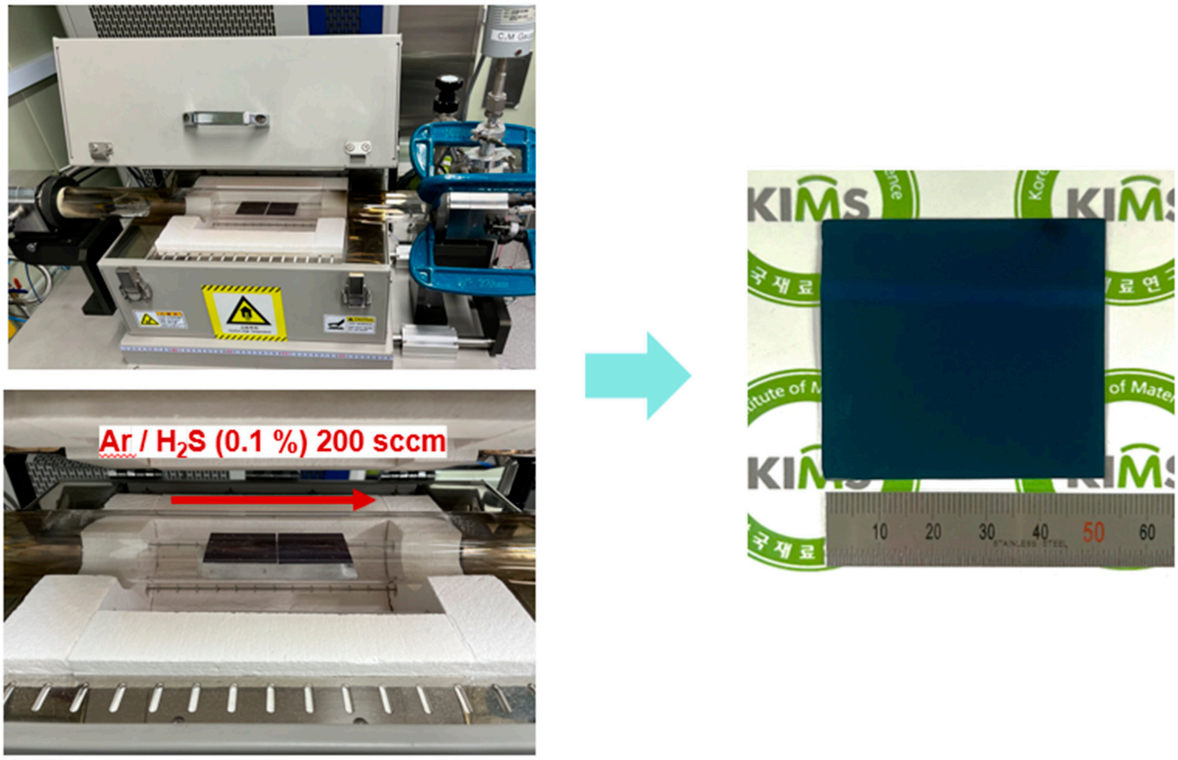

Figure S2. The image of sulfurization MoO_3 deposited on 2-inch scale SiO_2 (100nm)/Si substrate using thermal Chemical Vapor Deposition (CVD).

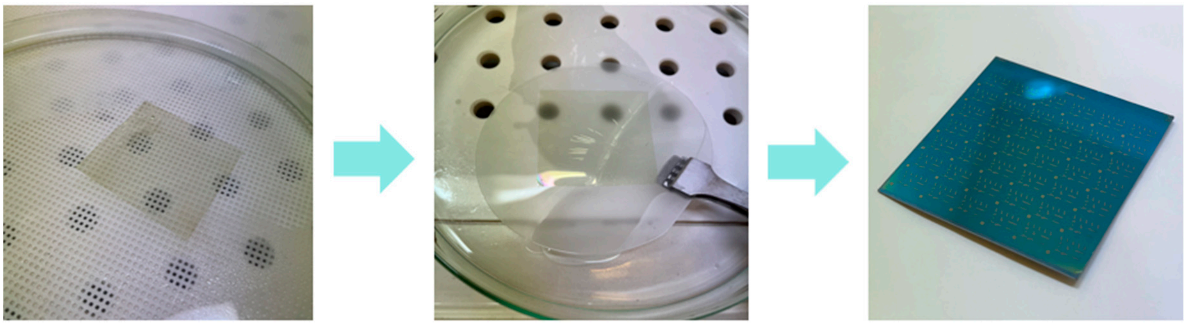

Figure S3. The image of separated MoS_2 film from the SiO_2 (100 nm)/Si substrate transferred on to another substrate which has been transferred to the gate dielectric layer

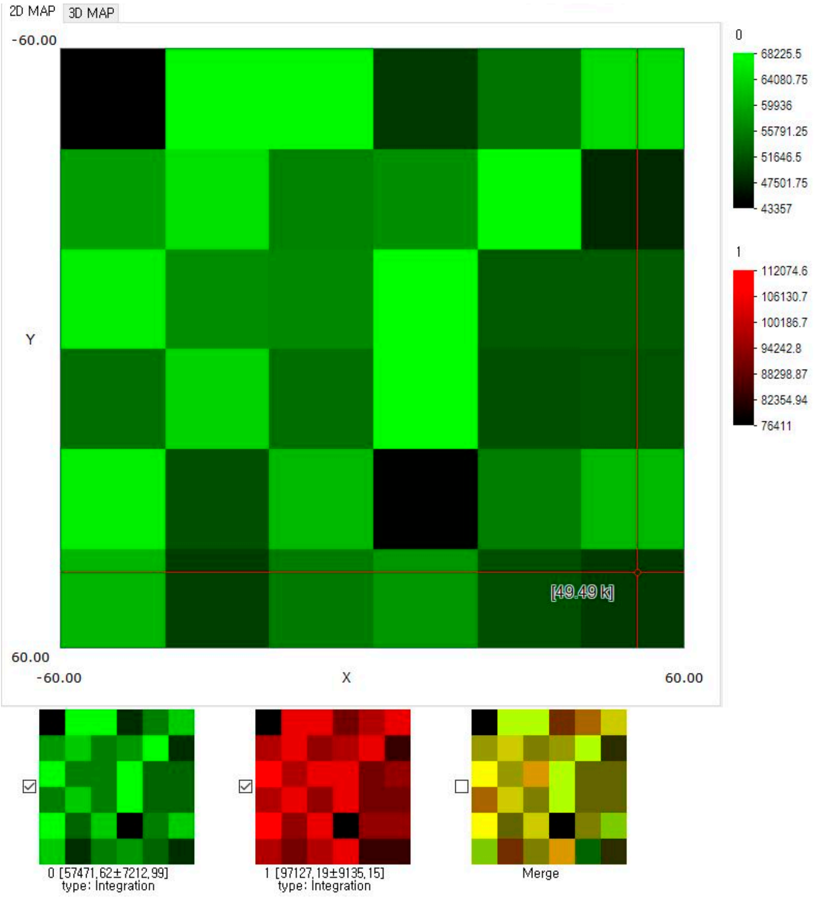

Figure S4. The actual Raman mapping image of a 2D-MoS_2 film. The mapping divides an area of 50 x 50 um^2 sections of 36 points, each with a step size of 10 μm.

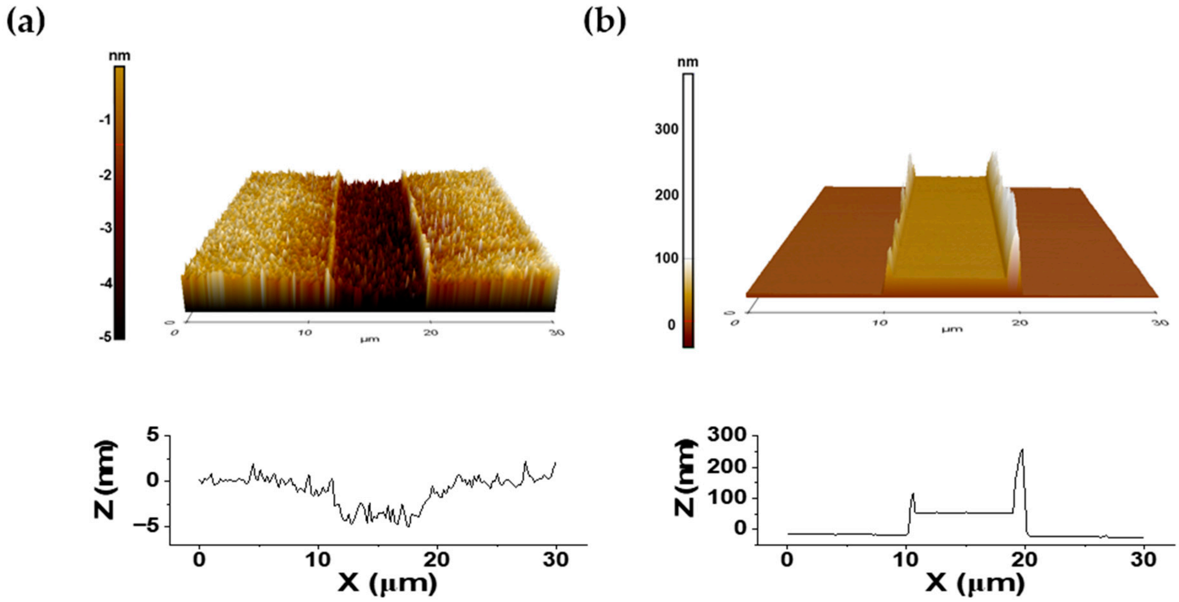

Figure S5. (a and b) Atomic force microscopy (AFM) images and height profiles of the sidewalls of buried-gate and local back-gate filled with gate metal.

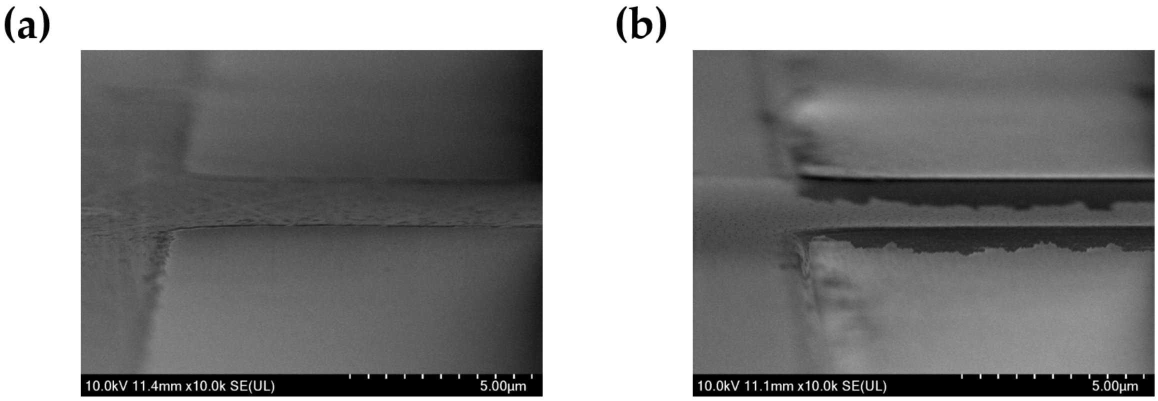

Figure S6. The scanning electron microscope (SEM) images of tilted gate surfaces of (a) buried-gate and (b) local back-gate. The buried gate is stably embedded in the etched SiO2 layer, displaying a clean edge shape. In contrast, the local back-gate surface has a sidewall at the edge that causes the deposited metal to roll out, resulting in a rough surface.

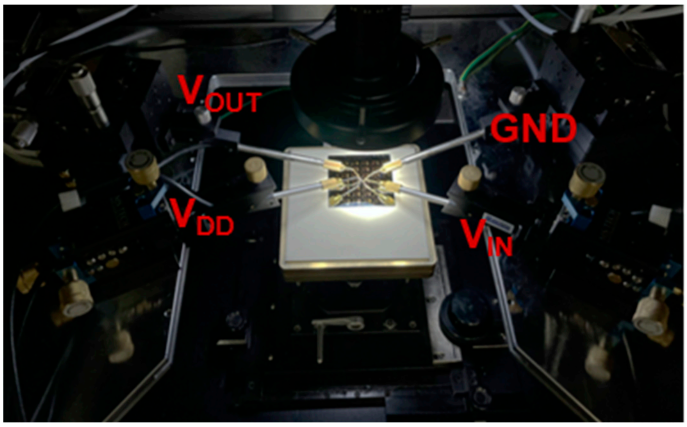

Figure S7. The image of electrical measurement of logic device using a semiconductor parameter analyzer system (Keithley-4200 SCS) at room temperature (R.T.).

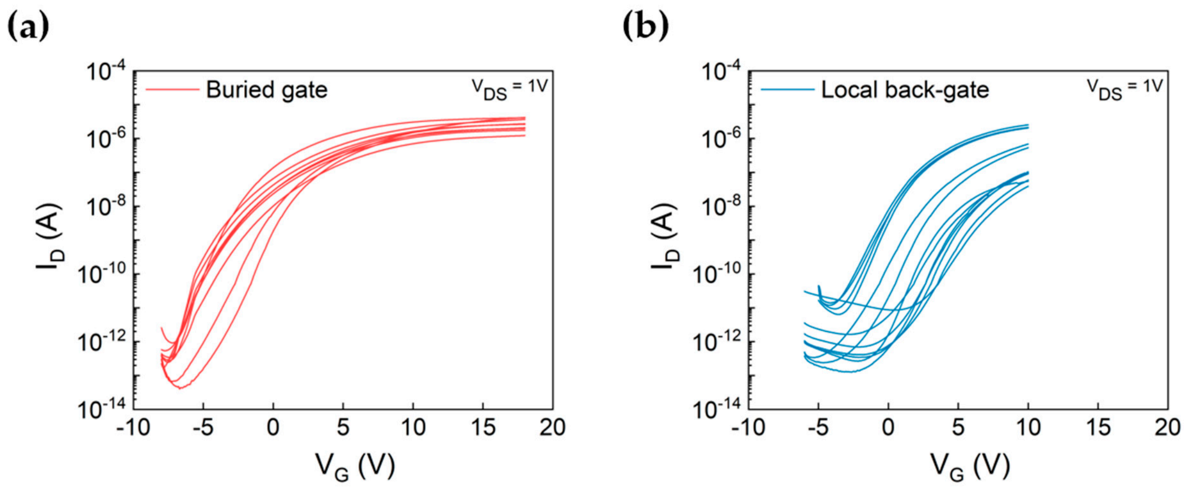

Figure S8. More data collected from each gate type of MoS_2-FETs. Each data shows transfer curves measured from 15 FETs which are randomly located on different positions of the substrate. (a) buried-gate, (b) local back-gate.

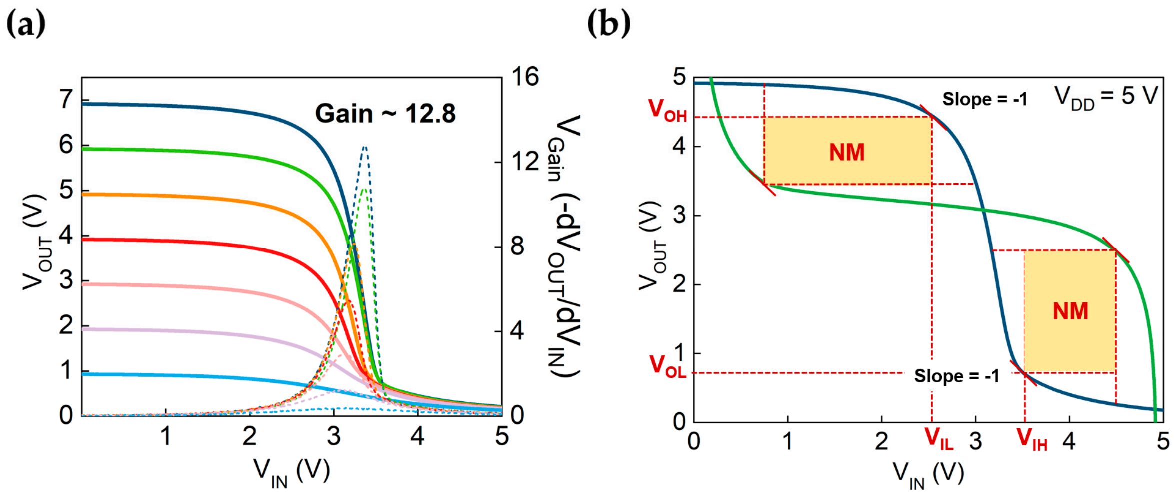

Figure S9. (a) Voltage Transfer Curve (VTC) of the local back-gate based inverter under supply V_{DD} from 1 to 7 V. The best performance of voltage gain is 12.8. (While the buried-gate is 17.67, the local back-gate shows lower value.) (b) Noise Margin (NM) properties of the local back-gate based inverter at V_{DD} = 5 V, the curve acquired by mirroring the Voltage Transfer Curve (VTC), and the position of slope equals to -1 marked. The inverter calculated NM_L and NM_H values of 1.56 V and 0.9 V, respectively.
